# Supplementary material for: Exploring access to HIV-related services and programmatic gaps for Men having Sex with Men (MSM) in rural India- a qualitative study
Source: PLoS One. 2023 May 4;18(5):e0284901. doi: 10.1371/journal.pone.0284901 (PMC10159193; doi:10.1371/journal.pone.0284901)
Supplement: Attachment — Submitted filename: MSM _review.pdf [file pone.0284901.s001.pdf]

Thank you for the opportunity to review this interesting paper. The authors conducted a qualitative study in four states of India to identify barriers and enablers for men who have sex with men in using national HIV programmes. using grounded theory the authors conductor range of interviews to identify common themes related to cultural barriers low visibility and health system challenges and linking MSM two required HIV prevention programmes. The paper provides important findings button into India that advance the literature in HIV prevention I have identified some comments below which I encourage the authors to address in order to refine the presentation of the results

Comment

- **Line 51,page 11:** The underlying assumption that MSM are driving HIV incidence in India is challenging considering the high rates of blood borne routes that predominate in the North East. In this context, can the authors provide additional details of the current hIV status In India Moreover the reference quoted ref(13) is a 2012 NACO report which does not indicate current status.
- The framing/rationale of the study aim, and context requires strengthening by highlighting important epidemiological and public health information from India in the background. HI prevalence among MSM needs to be situated first across overall HIV prevalence and incidence in India with reference to India's progress over the MDG to meet the global HIV 90-90-90 targets.
- **Lines 72: page 12**, Please provide an overview of India's HIV prevention national program in brief for international readers. Please explain what the link worker scheme is referenced in line 72.
- **In lines 85**, please say what the HSS HIV prevalence rates for the study sites are- providing key context is important here.
- **Lines 91,13:** While retaining confidentiality, please provide some description of who the community liaison and key population health leaders were.
- As part of the methods, the authors mention study areas however, can they please provide a break up of exact data collection numbers split across the study areas and across key respondents. Currently, the paper does not distinguish between the study areas despite known development, cultural and ethnical variables acting in each state which may differently impact MSM's engagement with the program

- Understanding the results requires reference to the specific study sites and areas from where data was collected, are the authors able to identify common themes relevant to individual states from where data was collected in order to provide meaningful recommendation to policy makers

- **General comment about references**
- 1.Where possible use newer sources of data/literature . Ref 13 for example is the 2012 NACO report which has since been updated
- 2.Please review the references for formatting issues. Currently, the author names and key headings for many references have errors for example reference 12(Trust IHA) is incorrect 16 has text formatting issues' 17 UNAIDS needs to be in full form, Ref 20 - text formatting errors.
- These are only some that I am sharing here for illustration, please review the full reference list for consistency
