# Supplementary material for: Exploring access to HIV-related services and programmatic gaps for Men having Sex with Men (MSM) in rural India- a qualitative study
Source: PLoS One. 2023 May 4;18(5):e0284901. doi: 10.1371/journal.pone.0284901 (PMC10159193; doi:10.1371/journal.pone.0284901)
Supplement: Attachment — Submitted filename: Point by point responses to reviewers 09 Feb 23.docx [file pone.0284901.s003.docx]

**Point by point responses to reviewer’s comments**

**Reviewer #1:**

**Comment**: Thank you for your attention to detail and the issues raised in my previous review of the original manuscript. You have satisfactorily addressed my previous comments. This paper addresses many important aspects of rural Indian HIV service gaps and societal underpinnings that require immediate attention. Congratulations on bringing such an important piece to international attention.

**Response**: Thank you. We are grateful.

**Reviewer #2:**

**Comment:** A much-improved version is submitted, which clarifies prior comments. Again this is a paper of great public health importance, and I would be pleased to see it published soon.

The current revision still requires language editing for clarity and adherence to an academic style guide. For example, please spell out numbers below 10 instead of using 4, 5 etc., as done in places.

**Response:** Thank you for your guidance. We have corrected the language editing for clarity adhering to APA academic style.

Page nos: 3, 8, 9, 11.16.17.19, 26, 28

Line nos: 29, 30,163, 166, 178, 188, 189, 238, 349, 357, 366, 378, 379,383, 427, 580, 625 (Line numbers as per track changed copy with all mark-up setting)

**Comment:** The second key comment is for authors to review the literature-results-discussion sections to provide better linkages in current programmatic gaps in the study sites that led to the study. I understand in the literature section prevalence of MSM with HIV. MSM is a factor as is anonymity and sociocultural issues in rural areas, but can the authors please review the literature section and provide clearer links (rationale) for the study related to rural areas given that NACO certainly has a rural component in place where health services are active. For example, you can contrast NACO’s success in rural areas in other states and perhaps state that the three study sites are also considered part of socio-economically disadvantaged states.

**Response:** We agree with the reviewer regarding NACO’s rural component being active. We have accordingly included the literature in the introduction section.

Page nos: 5, 6

Line nos: 83-86, 97-102 (Line numbers as per track changed copy with all mark-up setting)

We are thankful for this suggestion as it has provided strong rationale for including particular study sites.

**Comment:** As there is a large co-authorship for the paper, I would like the paper to be reviewed for clarity in the communication of the key messages across the three sections that I have identified above.

**Response:** As suggested we have circulated the revised version to all authors for their review and suggestions if any.

**Comment:** The conclusion also needs to be reworded as currently, it reads like a programmatic implications section which fits better under discussion. The authors may like to read the literature and go straight to discussion to see how the paper flows as a tip

**Response:** Thank you for your suggestions. We have shifted the conclusion points to discussion section. We have accordingly revised the conclusion.

Page no: 32-33

Line no: 732-746

**Reviewer #3:**

**Comment:** The revised manuscript addresses all my previous comments satisfactorily. In some places there are minor language errors, and therefore I recommend a thorough re-checking of the text, otherwise in terms of content I do not have any further suggestions.

**Response:** Thank you for reviewing the manuscript. We have carefully gone through the manuscript for English language editing and tried our best to remove any error.
